# Editorial: structural plasticity induced by drugs of abuse

**DOI:** 10.3389/fphar.2015.00088

**Published:** 2015-05-15

**Authors:** M. Foster Olive, Justin T. Gass

**Affiliations:** ^1^Behavioral Neuroscience Area, Department of Psychology, Arizona State UniversityTempe, AZ, USA; ^2^Department of Neurosciences, Medical University of South CarolinaCharleston, SC, USA

**Keywords:** addiction, plasticity, glutamate, neurotrophin, GABA, homer, oligodendrocyte, cytoskeleton

The roots of addiction are often attributed to the ability of repeated drug use to compromise proper functioning of the central nervous system. Drug-induced functional changes in the brain occur on many levels, including altered expression of specific genes via genomic and epigenetic mechanisms, induction of synaptic plasticity and other cellular adaptations, and volumetric changes in discrete brain regions (Luscher and Malenka, 2011). These alterations can be drug class-specific and occur in both neuronal and non-neuronal cell populations. Drug-induced plasticity, or “rewiring” of the brain contributes to the development, maintenance, and persistence of the addicted state (Kalivas and O'Brien, 2008). The goal of this Research Topic is to assimilate recent findings related to plasticity and structural alterations produced by drug of abuse in neurons, glia, and other cell types of the brain.

The mesolimbic dopamine system, originating in the ventral tegmental area (VTA) of the midbrain and projecting rostrally to the nucleus accumbens (NAc) and prefrontal cortex (PFC), mediates the acute reinforcing and incentive salience of abused drugs, as well as their aversive properties. As reviewed by Vashchinkina et al. (2014) mesolimbic dopamine neurons are highly regulated by intrinsic and extrinsic inhibitory GABAergic neurons. Vashchinkina et al. (2014) hypothesize that abused drugs induce structural and functional mesolimbic adaptations differentially via endogenous (e.g., THIP and neurosteroids) vs. exogenous (e.g., benzodiazepines and alcohol) modulators of GABA_A_ receptors, as well as via synaptic vs. extrasynaptic GABA_A_ receptors in the VTA. Collo et al. (2014) review evidence that drug-induced plasticity in mesolimbic dopaminergic neurons is mediated by common dopamine and brain-derived neurotrophic factor (BDNF) signaling pathways, specifically those recruiting MEK-ERK1/2, and PI3K-Akt-mTOR. Cadet and Bisagno (2014) review evidence for substantial plasticity in non-neuronal cell types in brain reward circuitry, including changes in astrocytic glutamate transporter expression, increases in pro-inflammatory cytokine production by microglia, and dysregulated oligodendrocytic myelin production. This latter topic is further elaborated on by Somkuwar et al. (2014) in their review of the proteoglycan neuron-glial antigen 2 (NG2), its expression by oligodendrocyte progenitor cells in the brain reward circuitry, and its interaction with stress-related neuromodulators. In addition, Bajo et al. (2015) provide evidence that interleukin-1β alters both basal and ethanol-facilitated GABAergic transmission in the central nucleus of the amygdala, part of the extended amygdala circuitry which is implicated in ethanol's central effects.

Drugs of abuse also alter the dynamics and microstructure of both dendrites and dendritic spines, which can be interpreted as cellular (mal)adaptations that reinforce the addiction cycle (Luscher and Malenka, 2011; Gipson et al., 2014). DePoy et al. (2014) show that repeated exposure of early adolescent mice to cocaine produces lasting reductions in orbitofrontal cortex dendritic arbor length and complexity and as well as impaired reversal learning. In addition, these investigators show that while mice carrying a heterozygous deletion of the actin cytoskeleton stabilizing protein p190RhoGAP exhibit enhanced vulnerability to cocaine-induced hyperlocomotion, orbitofrontal dendritic complexity in cocaine-naïve mice is intact. These findings suggest that orbitofrontal dendrite structure is impacted by repeated cocaine during adolescence, but pre-existing structural dendritic deficiencies do not account for increased behavioral sensitivity to this drug.

Glutamate is the predominant excitatory amino acid in the central nervous system and mediates both normal and maladaptive cellular plasticity. Several articles in this Research Topic provide novel findings on the role of glutamate in addictive processes. Weiland et al. (2015) demonstrate that antibiotics with the glutamate and GABA modulating properties (ceftriaxone and cefazolin, respectively) attenuate cue-primed reinstatement of alcohol-seeking. Griffin et al. (2015) demonstrate that chronic intermittent ethanol exposure results in increased extracellular levels of glutamate in the NAc in ethanol-dependent mice, but these effects are not a result of locally dysregulated sodium-dependent and independent glutamate transporter function, suggesting that extrinsic corticostriatal glutamatergic pathways may contribute to this hyperglutamatergic state. Finally, McGuier et al. (2015) show that deletion of the excitatory postsynaptic scaffolding protein Homer2 is associated with an increased density of long, thin dendritic spines in NAc core medium spiny neurons, yet unexpectedly these structural modifications are not modified by repeated alcohol exposure.

Together, this body of work indicates complex interactions between drugs of abuse, endogenous neuromodulators and their signaling targets, and the mechanisms underlying the functional and structural plasticity in the brain. Research into this complexity is only in its infancy, and needs to be pursued at multiple levels in order to better understand and treat addictive disorders.

## Conflict of interest statement

The authors declare that the research was conducted in the absence of any commercial or financial relationships that could be construed as a potential conflict of interest.
